# HEALTH DIMENSIONS AS PREDICTORS OF HOME MODIFICATION IMPLEMENTATION: A LONGITUDINAL ANALYSIS OF NHATS DATA

**DOI:** 10.1093/geroni/igae098.0007

**Published:** 2024-12-31

**Authors:** Bernard A Steinman, Mengzhao Yan, Jon Pynoos

**Affiliations:** University of Wyoming, Laramie, Wyoming, United States; University of Southern California, Los Angeles, California, United States; University of Southern California, Los Angeles, California, United States

## Abstract

A significant proportion of older Americans experience chronic conditions, functional impairments, participation limitations, activities of daily living (ADL) and instrumental activities of daily living (IADL) disabilities that could impact their capacity to live independently in their homes. Home modifications (HM) implemented to promote safety and access can support aging in place by improving environmental fit with health characteristics of older individuals. We conceptualized health dimensions using the International Classification of Functioning, Disability, and Health (ICF) to estimate risk profiles for implementing shower/tub and toilet HMs. We analyzed data from the National Health and Aging Trends Study (NHATS) using Cox regression, to examine associations between each ICF health dimension (i.e., personal characteristics, chronic conditions, functional impairments, social participation, ADL and IADL disability) and the probability of community dwelling older adults implementing HMs over a five-year period (2011 to 2015). Results indicated that being older age, married, having more functional impairments, greater social participation, and greater ADL and IADL disabilities are each independently associated with a higher probability of acquiring both types of HM. For example, each additional functional limitation was associated with a 12% increased risk of implementing toilet modifications and a 4% increased risk of implementing shower/tub modifications. Additionally, compared to non-Hispanic Whites, Hispanics are less likely to implement either type of HM. Implications of this study are that older individuals choose to implement HMs in response to different need profiles and that some age-related changes may be more impactful in decision making than others.

